# Prevalence and Perioperative Implications of Cardiovascular Comorbidities in Non-Cardiac Surgery: A Three-Year Retrospective Cohort Study

**DOI:** 10.3390/medicina62071280

**Published:** 2026-07-03

**Authors:** Bogdan-Florin Godje, Sergiu-Ciprian Matei, Ana-Maria Ungureanu, Daniel Malița, Ovidiu Ghirlea, Andrei Florin Părău, Marius-Sorin Murariu

**Affiliations:** 1 Department of Doctoral Studies, “Victor Babeș” University of Medicine and Pharmacy, Eftimie Murgu Square, No. 2, 300041 Timișoara, Romania; bogdan.godje@umft.ro; 2 1’st Surgical Department, “Pius Brînzeu” Emergency County Hospital Timișoara, 300723 Timișoara, Romania; matei.sergiu@umft.ro (S.-C.M.); ovidiu.ghirlea@umft.ro (O.G.); parau.andrei@umft.ro (A.F.P.); murariu.marius@umft.ro (M.-S.M.); 3 Abdominal Surgery and Phlebology Research Center, “Victor Babes” University of Medicine and Pharmacy, Eftimie Murgu Square, No. 2, 300041 Timișoara, Romania; 4 Department XV, Clinic of Radiology and Medical Imaging, “Victor Babes” University of Medicine and Pharmacy, Eftimie Murgu Square, No. 2, 300041 Timisoara, Romania; malita.daniel@umft.ro; 5 Department of Radiology and Medical Imaging, “Pius Brinzeu” Emergency County Hospital, 300723 Timisoara, Romania

**Keywords:** cardiovascular comorbidity, non-cardiac surgery, hypertension, perioperative management, anticoagulation, ESC guidelines

## Abstract

*Background and Objectives*: Cardiovascular diseases (CVDs) are the leading cause of global morbidity and mortality, and significantly influence perioperative risk in non-cardiac surgery. This study aimed to evaluate the prevalence of cardiovascular comorbidities and their immediate impact on perioperative management in a tertiary surgical center. *Materials and Methods*: A retrospective cohort study was conducted including all adult patients undergoing elective or emergency non-cardiac surgery between January 2022 and December 2024. Demographic data, primary diagnoses, cardiovascular comorbidities, chronic medication, and perioperative management modifications were analyzed. Descriptive statistical analysis was performed using SPSS v16. *Results*: A total of 5716 patients were included. Cardiovascular comorbidities were identified in 2157 patients (37.37%). Hypertension (HTN) was the most prevalent condition (33.37%). Surgical delay due to anticoagulation occurred in 4.16% of cases, postponement due to cardiovascular instability in 1.27%, and surgery was contraindicated in 0.50%. *Conclusions*: Cardiovascular comorbidities are highly prevalent among patients undergoing non-cardiac surgery and frequently require modification of perioperative management strategies. Further studies incorporating postoperative outcomes are needed to better define their impact on surgical prognosis. Structured cardiovascular risk assessment aligned with ESC (European Society of Cardiology) guidelines appears clinically justified, although future outcome-based studies are required.

## 1. Introduction

Cardiovascular diseases (CVDs) remain the leading cause of morbidity and mortality worldwide [1,2]. The increasing prevalence of hypertension, ischemic heart disease, heart failure, and arrhythmias has significant implications for patients undergoing surgical interventions [3,4,5]. As surgical populations age and multimorbidity become more frequent, cardiovascular comorbidities increasingly influence perioperative risk stratification.

In general surgery departments, a substantial proportion of admissions are related to digestive oncologic pathology, abdominal wall defects, biliary disease, and thyroid disorders [6,7,8,9,10]. Digestive malignancies represent a major component of surgical workload and are associated with systemic inflammatory and prothrombotic states [11,12,13]. Cancer itself increases the risk of thromboembolic events, a risk that may be further amplified in the presence of cardiovascular comorbidities [14,15].

Abdominal wall hernias and incisional hernias are common surgical conditions that significantly impact patient quality of life and frequently require operative management [6,7,8,9]. Likewise, acute and chronic cholecystitis remain among the most frequent causes of surgical admission worldwide [10,11]. These high-prevalence pathologies often affect middle-aged and elderly patients, a population in which cardiovascular comorbidities are common.

The perioperative period is characterized by hemodynamic stress, inflammatory activation, and anesthetic-induced myocardial depression [16,17,18]. In patients with pre-existing cardiovascular disease, these physiological stressors may precipitate decompensation or ischemic events. Management of chronic anticoagulant and antiplatelet therapy is particularly challenging in surgical patients. Current guidelines recommend individualized perioperative strategies to balance thromboembolic and bleeding risks [19,20,21,22,23,24]. These considerations are especially relevant in oncologic patients, who already exhibit an increased risk of venous thromboembolism [14,15].

Despite the recognized importance of cardiovascular risk assessment in non-cardiac surgery, real-world data from Eastern European surgical populations remain limited. The aim of this study was therefore to evaluate the prevalence of cardiovascular comorbidities in a large tertiary surgical center and to assess their immediate impact on perioperative management.

The concept of perioperative medicine has gained increasing importance in recent years, particularly in aging surgical populations with multiple chronic diseases. Modern perioperative care extends beyond the surgical act itself and involves comprehensive preoperative risk assessment, optimization of chronic comorbidities, individualized anesthetic planning, and postoperative surveillance. In this context, cardiovascular evaluation represents one of the cornerstones of perioperative management, especially in tertiary surgical centers treating oncologic and complex abdominal pathology.

From a practical perspective, the perioperative relevance of cardiovascular disease is not limited to postoperative adverse events. Even before surgery, chronic antithrombotic therapy, hemodynamic instability, the need for additional cardiological evaluation, and anesthetic risk reassessment may alter surgical timing, operating room planning, bed allocation, and interdisciplinary communication. These aspects are particularly important in high-volume surgical departments, where a relatively small proportion of delayed, postponed, or contraindicated procedures may translate into a substantial organizational impact over several years.

## 2. Materials and Methods

### 2.1. Study Design and Setting

This retrospective cohort study was conducted at the First Department of Surgery, “Pius Brînzeu” Emergency County Hospital, Timișoara, Romania, a tertiary referral center. The study analyzed all consecutive patients admitted for surgical intervention between January 2022 and December 2024.

The study followed a retrospective observational design. The workflow consisted of identifying all eligible adult patients admitted for non-cardiac surgery during the predefined three-year interval, excluding records with insufficient clinical information, extracting demographic, diagnostic, cardiovascular, medication-related, and perioperative management variables, coding cardiovascular comorbidities and perioperative management modifications, and performing descriptive analysis of the overall cohort and cardiovascular subgroup. No experimental intervention was performed; the objective was to describe real-world perioperative practice in a tertiary surgical setting.

### 2.2. Study Population

All adult patients (≥18 years) undergoing elective or emergency non-cardiac surgical procedures during the study period were included. No restriction was applied based on surgical diagnosis. Patients with incomplete medical records were excluded from analysis.

The inclusion of consecutive patients was intended to minimize selection bias and to reflect the routine case-mix of the department. Patients were not excluded on the basis of age category, surgical diagnosis, urgency of admission, or type of non-cardiac operation, provided that the medical record contained the variables required for endpoint assessment.

### 2.3. Data Collection

Data were extracted from electronic and paper-based medical records. The following variables were collected: demographic data (age, sex, area of residence); primary surgical diagnosis; type of surgical intervention; cardiovascular comorbidities (including hypertension, valvular heart disease, heart failure, atrial fibrillation, atrioventricular block, history of myocardial infarction, and other documented cardiac conditions); cardiovascular risk factors (obesity, dyslipidemia, chronic smoking)*;* chronic cardiovascular medication (beta-blockers, ACE inhibitors, angiotensin receptor blockers, statins, antiplatelet agents, anticoagulants); and perioperative management modifications (surgical delay due to anticoagulation, postponement due to cardiovascular decompensation, anesthetic contraindication).

Cardiovascular comorbidities were defined based on documented diagnoses in the patients’ medical records at the time of admission.

To improve reproducibility, variables were extracted using a predefined data collection structure based on information available in admission notes, surgical records, anesthesiology assessments, cardiology consultations, and discharge documents. When more than one cardiovascular diagnosis was documented in the same patient, each condition was counted separately for condition-specific prevalence, whereas each patient was counted only once when estimating the overall cardiovascular comorbidity burden. Chronic cardiovascular medication was recorded when documented in the medical chart at admission or in the medication history.

Surgical procedures were characterized according to the primary surgical diagnosis and the clinical context documented in the medical record. Elective procedures were defined as planned interventions performed after standard preoperative assessment, whereas emergency procedures were defined as interventions performed during urgent admission or because of acute surgical pathology. Procedure complexity was interpreted clinically according to the extent of surgery, anesthetic requirements, expected physiological stress, and anticipated need for postoperative monitoring. Because the retrospective records did not uniformly contain standardized coding for surgical complexity across all cases, this variable was used for contextual interpretation rather than formal subgroup modeling.

### 2.4. Study Endpoints

The primary endpoint was the prevalence of cardiovascular comorbidities among patients undergoing non-cardiac surgery.

Secondary endpoints included the frequency of perioperative surgical delay due to anticoagulant therapy, postponement of surgery due to cardiovascular decompensation or need for additional investigations, and contraindication of surgical intervention due to high anesthetic cardiovascular risk.

For the purposes of this study, perioperative management modification was defined as any documented alteration of the planned surgical pathway attributable to cardiovascular disease, cardiovascular risk factors, or cardiovascular treatment. The three categories analyzed (surgical delay due to anticoagulant therapy, postponement due to cardiovascular instability or additional cardiologic assessment, and contraindication due to excessive anesthetic cardiovascular risk) were considered mutually exclusive. Each patient was assigned to a single category according to the primary documented reason for modification of surgical management.

These outcomes were analyzed both individually and as a composite indicator of cardiovascular-related perioperative management modification. The composite indicator was intended to capture clinically relevant changes in the planned surgical pathway and should not be interpreted as a postoperative morbidity endpoint.

### 2.5. Statistical Analysis

Statistical analyses were performed using the Statistical Package for Social Sciences (SPSS), version 16.0 (SPSS Inc., Chicago, IL, USA).

Continuous variables are presented as mean ± standard deviation, whereas categorical variables are expressed as absolute numbers and percentages. Given the descriptive objective of the study, the analysis focused on characterizing cardiovascular comorbidities, associated cardiovascular risk factors, chronic cardiovascular medication, and cardiovascular-related perioperative management modifications. Percentages for baseline demographic and perioperative management variables were calculated using the entire study cohort (*n* = 5716) as denominator, whereas medication percentages were calculated among patients with documented cardiovascular comorbidities (*n* = 2157). Because complete patient-level datasets required for reliable subgroup comparisons and multivariable modeling were not uniformly available from the retrospective records, no adjusted regression models were performed. The results should therefore be interpreted as estimates of disease burden and management impact rather than causal associations.

### 2.6. Ethical Considerations

The study was conducted in accordance with the principles of the Declaration of Helsinki and approved by the “Pius Brînzeu” University Clinical Hospital Timisoara Ethical Committee (Timisoara, Romania; REC no. 122, approved on 16 April 2025).

## 3. Results

### 3.1. Patient Characteristics

A total of 5716 patients underwent non-cardiac surgical procedures between January 2022 and December 2024. The annual distribution was as follows: 1804 patients in 2022, 1939 in 2023, and 1973 in 2024.

The study population included 2757 women (48.2%) and 2959 men (51.8%), demonstrating a relatively balanced sex distribution. Regarding residence, 3069 patients (53.7%) originated from urban areas, while 2647 (46.3%) were from rural settings.

The mean age of the overall cohort was 54.9 ± 12.7 years (range 18–97 years). A higher concentration of patients was observed in the fifth to eighth decades of life.

Patient characteristics are summarized in Table 1.

Patients older than 50 years represented most of the study population, reflecting the predominance of middle-aged and elderly individuals in general surgical practice. This age distribution is clinically relevant, as cardiovascular diseases and associated metabolic disorders become increasingly prevalent with advancing age.

The relatively balanced annual distribution of admissions over the three-year interval suggests that the cohort reflects a stable institutional surgical workload rather than a single-year fluctuation. This supports the use of the cohort as a real-world estimate of cardiovascular comorbidity burden in routine surgical practice.

### 3.2. Primary Surgical Diagnoses and Procedures

The most frequent primary admission diagnoses were: digestive cancers—15.57% (*n* = 897 patients); acute cholecystitis—12.92% (*n* = 739 patients); nodular thyroid goiter—12.8% (*n* = 732 patients); abdominal wall pathology (hernias and incisional hernias)—12.08% (*n* = 691 patients); varicose veins—3.11% (*n* = 178 patients); and other pathologies, including surgical infections, lower limbs gangrene, cutaneous and subcutaneous tumors, etc.—43.36% (*n* = 2479 patients).

Both elective and emergency procedures were included in the analysis.

This case-mix reflects the heterogeneity of a tertiary general surgery department, including oncologic, biliary, endocrine, abdominal wall, vascular, infectious, and soft-tissue pathology. Such heterogeneity is clinically relevant because cardiovascular comorbidities may influence surgical planning differently across elective and emergency settings, particularly through anticoagulant management, hemodynamic optimization, and anesthetic risk assessment.

### 3.3. Prevalence of Cardiovascular Comorbidities

**Figure 1:**
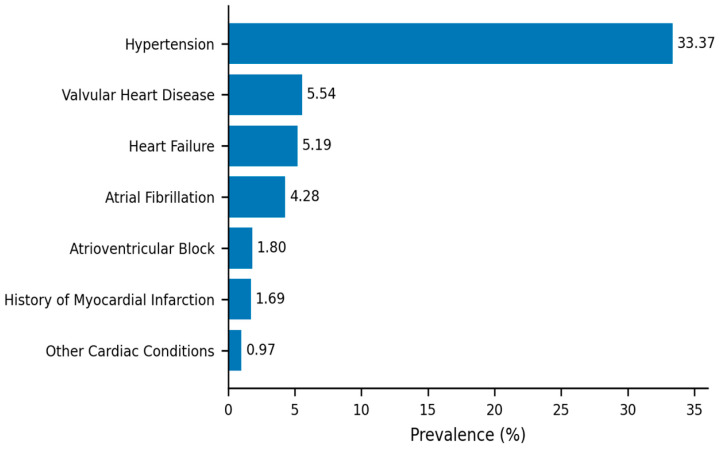
Distribution of cardiovascular comorbidities in the study population.

Cardiovascular comorbidities were identified in 2157 patients, representing 37.37% of the total cohort (Figure 1).

The distribution of cardiovascular conditions was as follows—see Table 2.

• Hypertension—33.37% (*n* = 1908 patients)
• Valvular heart disease—5.54% (*n* = 317 patients)
• Heart failure—5.19% (*n* = 297 patients)
• Atrial fibrillation—4.28% (*n* = 245 patients)
• Atrioventricular block—1.8% (*n* = 103 patients)
• History of myocardial infarction—1.69% (*n* = 97 patients)
• Other cardiac conditions (including other arrhythmias, prior myocarditis, ventricular septal defect, etc.)—0.97% (56 patients)

The predominance of hypertension among cardiovascular comorbidities is consistent with current epidemiological data identifying hypertension as the most prevalent chronic cardiovascular condition worldwide. Additionally, the frequent coexistence of hypertension with diabetes mellitus and other metabolic disorders reflects the complex multimorbid profile commonly encountered in surgical patients.

Hypertension was frequently associated with additional comorbidities, being combined with other cardiovascular or metabolic disorders in 1659 cases (86.94% of hypertensive patients). The most common association was hypertension and diabetes mellitus (621 patients).

Patients with cardiovascular comorbidities represented 37.37% of the cohort and were predominantly distributed in older age groups, reflecting the known age-dependent increase in cardiovascular disease prevalence. Although detailed subgroup statistical comparisons were limited by the retrospective design, the presence of cardiovascular pathology was consistently associated with increased complexity of perioperative management.

### 3.4. Cardiovascular Risk Factors

The most frequently documented cardiovascular risk factors were obesity, dyslipidemia, and chronic smoking. These factors were commonly associated with established cardiovascular disease, further contributing to perioperative risk stratification complexity.

### 3.5. Chronic Cardiovascular Medication

Among patients with cardiovascular disease (*n* = 2157), the distribution of chronic cardiovascular medication is presented in Table 3.

### 3.6. Perioperative Impact of Cardiovascular Comorbidities

Cardiovascular disease had a measurable impact on surgical management.

Surgical intervention was delayed in 238 patients (4.16%) due to chronic anticoagulant therapy requiring temporary discontinuation or bridging therapy. In 37 patients (1.27%), surgery was postponed due to cardiovascular decompensation or the need for additional cardiological evaluation and treatment optimization. In 29 patients (0.5%), surgical intervention was contraindicated due to severe cardiovascular pathology associated with major anesthetic risk (Table 4).

**Figure 2:**
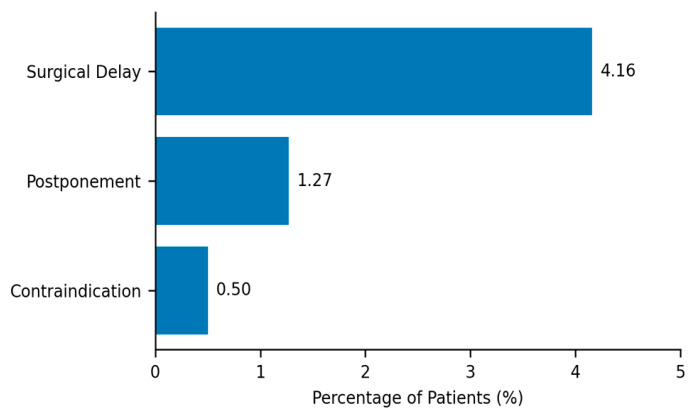
Perioperative impact of cardiovascular comorbidities.

Overall, perioperative management was altered in approximately 5.9% of patients due to cardiovascular-related factors, highlighting the clinical relevance of these comorbidities in routine surgical practice (Figure 2).

The high prevalence of beta-blocker and ACE inhibitor therapy reflects the substantial burden of chronic cardiovascular disease in the analyzed population. Moreover, the significant proportion of patients receiving anticoagulant or antiplatelet therapy highlights the complexity of perioperative therapeutic management in modern surgical practice.

The categories of perioperative management modification were mutually exclusive and were assigned according to the primary documented cardiovascular-related reason for alteration of the planned surgical intervention.

Although the proportion of patients requiring perioperative management modification was limited at the cohort level, the absolute number of affected patients was clinically meaningful. Over the three-year period, 304 patients required a cardiovascular-related change in the planned surgical pathway, emphasizing the organizational relevance of cardiovascular assessment in high-volume surgical services.

## 4. Discussion

The present study demonstrates that more than one-third of patients undergoing non-cardiac surgery had documented cardiovascular comorbidities. This finding is particularly relevant in the context of modern general surgery, where a substantial proportion of cases involve digestive malignancies, abdominal wall pathology, and biliary disease [15,16,17,18,19,20].

A major strength of the present study is the large cohort size and the inclusion of consecutive patients undergoing a broad spectrum of non-cardiac surgical procedures in a tertiary referral center. Unlike studies focused on selected surgical populations, our analysis provides a comprehensive overview of the cardiovascular comorbidity burden encountered in routine surgical practice. Furthermore, data from Eastern European surgical populations remain relatively limited, highlighting the relevance of these findings for regional perioperative risk assessment and healthcare planning.

Digestive cancers represent a significant surgical burden worldwide [20,21,22]. Malignancy is associated with a hypercoagulable state and increased incidence of perioperative venous thromboembolism [23,24]. When combined with hypertension, atrial fibrillation, or ischemic heart disease, this creates a complex perioperative risk profile requiring interdisciplinary management.

Similarly, abdominal wall hernias and incisional hernias, considered low-risk procedures, frequently affect patients with multiple comorbidities [15,16,17,18].

One of the key findings of this study is the measurable impact of chronic anticoagulant therapy on surgical scheduling. Current recommendations from the American College of Cardiology and the American College of Surgeons emphasize individualized perioperative anticoagulation management [25,26]. Bridging therapy with heparin and careful timing of drug discontinuation are essential to minimize both thromboembolic and bleeding risks [27,28].

Importantly, the perioperative management modifications analyzed in the present study represent distinct clinical scenarios rather than a single homogeneous outcome. Delays related to anticoagulant management, postponements caused by cardiovascular instability, and contraindications resulting from excessive anesthetic risk reflect different mechanisms through which cardiovascular disease may influence surgical care. Nevertheless, all three outcomes share a common characteristic: they necessitate modification of the initially planned surgical pathway and may contribute to increased healthcare resource utilization.

Anesthetic management also plays a critical role. Volatile anesthetics and other agents may induce myocardial depression and hemodynamic instability, particularly in patients with underlying cardiac dysfunction [16,17,29]. The postponement or contraindication of surgery observed in our cohort underscores the importance of thorough preoperative cardiovascular evaluation.

Although the present study focused primarily on the prevalence of cardiovascular comorbidities and their influence on perioperative management, the clinical significance of these findings extends beyond the preoperative period. Cardiovascular disease is a well-established risk factor for postoperative complications, including myocardial infarction, heart failure exacerbation, arrhythmia, cerebrovascular events, prolonged hospitalization, and increased mortality. Consequently, the high prevalence of cardiovascular pathology observed in our cohort highlights the potential burden of postoperative adverse events in surgical populations and reinforces the importance of comprehensive perioperative cardiovascular assessment.

Future prospective multicenter studies incorporating postoperative outcomes and long-term follow-up would provide additional insight into the prognostic implications of cardiovascular comorbidities in non-cardiac surgery.

Another important finding of our study is the substantial burden of cardiovascular risk factors among surgical patients. Obesity, dyslipidemia, and chronic smoking were frequently associated with established cardiovascular disease, contributing to increased perioperative risk. The high prevalence of hypertension observed in our cohort is consistent with global epidemiological data identifying hypertension as the most common cardiovascular condition worldwide. Given its association with hemodynamic instability, myocardial ischemia, and cerebrovascular complications, appropriate blood pressure optimization remains a key component of perioperative management. These findings further support the importance of interdisciplinary collaboration between surgeons, anesthesiologists, and cardiologists in the preoperative assessment and optimization of patients with complex cardiovascular profiles.

Finally, the results of the present study emphasize the importance of implementing standardized cardiovascular screening pathways for patients undergoing non-cardiac surgery. Systematic assessment using validated tools such as the Revised Cardiac Risk Index or guideline-based evaluation algorithms may improve identification of high-risk patients and facilitate individualized perioperative management strategies [25,26,27,28].

The potential applications of these findings are primarily organizational and clinical. At the institutional level, the results support the development of structured preoperative screening checklists, early identification of patients receiving antiplatelet or anticoagulant therapy, and predefined referral pathways for cardiology and anesthesiology assessment. In daily practice, such measures may help anticipate surgical delays, improve scheduling efficiency, and facilitate individualized perioperative planning, particularly in patients undergoing oncologic or complex abdominal procedures.

The present study has several limitations that should be acknowledged. First, its retrospective design may introduce selection bias and limit the ability to establish causal relationships between cardiovascular comorbidities and perioperative outcomes. Second, the study was conducted in a single tertiary center, which may affect the generalizability of the results to other healthcare settings or populations. Third, postoperative outcomes and long-term follow-up data were not included, preventing a comprehensive assessment of the prognostic impact of cardiovascular comorbidities beyond the immediate perioperative period. Additionally, the reliance on medical records may have led to underreporting or misclassification of certain comorbidities and risk factors. Future prospective, multicenter studies are warranted to validate these findings and to better define the long-term clinical implications. Fourth, standardized variables such as American Society of Anesthesiologists class, Revised Cardiac Risk Index components, frailty markers, operative duration, and uniformly coded surgical complexity were not consistently available and therefore could not be incorporated into adjusted analyses. These limitations should be considered when interpreting the reported perioperative management modifications.

Future research should prospectively collect patient-level variables, including surgical urgency, surgical complexity, anesthetic risk scores, cardiovascular risk indices, antithrombotic therapy details, perioperative biomarkers, postoperative cardiovascular complications, bleeding events, intensive care unit admission, length of hospital stay, readmissions, and mortality. Such data would allow multivariable modeling and help determine whether structured screening pathways translate into improved clinical outcomes.

Although this study did not assess postoperative outcomes, the high prevalence of cardiovascular pathology supports the need for standardized cardiovascular screening in surgical departments, particularly in tertiary referral centers managing complex oncologic and abdominal pathology.

An additional limitation is the lack of access to complete patient-level datasets for secondary analyses. Consequently, subgroup comparisons between patients with and without cardiovascular comorbidities and multivariable modeling of perioperative management modifications could not be performed. Future prospective studies incorporating standardized electronic datasets would allow more detailed statistical assessment of independent predictors of perioperative outcomes.

From a clinical perspective, the findings of this study highlight the importance of systematic cardiovascular assessment and optimization in patients undergoing non-cardiac surgery. Early identification of cardiovascular comorbidities and risk factors may support perioperative planning, including individualized management of anticoagulant therapy, optimization of hemodynamic status, and appropriate anesthetic strategies. Standardized protocols aligned with ESC guidelines may improve the consistency of risk identification and surgical planning; however, the present data do not establish that such protocols reduce postoperative complications or improve prognosis. These strategies are particularly relevant in tertiary referral centers managing a high proportion of oncologic and complex surgical cases.

## 5. Conclusions

Cardiovascular comorbidities were highly prevalent among patients undergoing non-cardiac surgery, affecting more than one-third of individuals in this large retrospective cohort.

Arterial hypertension was the most common cardiovascular condition and was frequently associated with additional metabolic and cardiac disorders.

Cardiovascular disease and cardiovascular-related therapies had a measurable impact on perioperative management, leading to surgical delay, postponement, or contraindication in a subset of patients.

These findings support the importance of careful cardiovascular assessment in patients undergoing non-cardiac surgery, particularly in tertiary centers managing complex surgical cases. However, as postoperative outcomes were not analyzed, this study does not establish whether standardized screening protocols reduce complications or improve clinical prognosis. Future prospective studies including postoperative morbidity, mortality, length of hospital stay, and readmission data are required to clarify this issue.

## Figures and Tables

**Table 1:** Demographic and Baseline Characteristics of the Study Population (*n* = 5716).

Variable	*n* (%)/Mean ± SD
**Total** **patients**	5716
**Sex**	
Female	2757 (48.2%)
Male	2959 (51.8%)
**Residence**	
Urban	3069 (53.7%)
Rural	2647 (46.3%)
**Age (years)**	
Mean ± SD	54.9 ± 12.7
Range	18–97
**Year of admission**	
2022	1804 (31.6%)
2023	1939 (33.9%)
2024	1973 (34.5%)

**Table 2:** Distribution of Cardiovascular Comorbidities in the Study Population.

Cardiovascular Condition	*n* (%)
Hypertension	1908 (33.37%)
Valvular heart disease	317 (5.54%)
Heart failure	297 (5.19%)
Atrial fibrillation	245 (4.28%)
Atrioventricular block	103 (1.80%)
History of myocardial infarction	97 (1.69%)
Other cardiac conditions	56 (0.97%)

**Table 3:** Chronic Cardiovascular Medication Among Patients with Cardiovascular Comorbidities (*n* = 2157).

Medication Class	*n* (%)
Beta-blockers	1434 (66.5%)
ACE inhibitors	1238 (57.4%)
Statins	654 (30.3%)
Antiplatelet agents	505 (23.4%)
Angiotensin receptor blockers (ARBs)	485 (22.5%)
Oral anticoagulants	451 (20.9%)

**Table 4:** Perioperative Impact of Cardiovascular Comorbidities.

Outcome	*n* (%)
Surgical delay (anticoagulation management)	238 (4.16%)
Postponement (cardiovascular instability)	37 (1.27%)
Contraindication (high anesthetic risk)	29 (0.50%)
**Total** **perioperative impact**	**304 (5.93%)**

## Data Availability

The original contributions presented in this study are included in the article. Further inquiries can be directed to the corresponding author.
